# A Bijective Image Encryption System Based on Hybrid Chaotic Map Diffusion and DNA Confusion

**DOI:** 10.3390/e22020180

**Published:** 2020-02-05

**Authors:** Dalia H. ElKamchouchi, Heba G. Mohamed, Karim H. Moussa

**Affiliations:** 1 Information Technology, College of Computer and Information Sciences, Princess Nourah Bint Abdulrahman University, Riyadh 11671, Saudi Arabia; dhelkamchouchi@pnu.edu.sa; 2 Electrical Department, College of Engineering, Alexandria Higher Institute of Engineering and Technology, Alexandria 21421, Egypt; 3 Electrical Department, College of Engineering, Princess Nourah Bint Abdulrahman University, Riyadh 11671, Saudi Arabia; 4 Electrical Department, College of Engineering, Horus University-Egypt, New Damietta 34518, Egypt; Khassan@horus.edu.eg

**Keywords:** hybrid chaotic, image, encryption, decryption, secured, communications, DNA

## Abstract

Modern multimedia communications technology requirements have raised security standards, which allows for enormous development in security standards. This article presents an innovative symmetric cryptosystem that depends on the hybrid chaotic Lorenz diffusion stage and DNA confusion stage. It involves two identical encryption and decryption algorithms, which simplifies the implementation of transmitting and receiving schemes of images securely as a bijective system. Both schemes utilize two distinctive non-consecutive chaotic diffusion stages and one DNA scrambling stage in between. The generation of the coded secret bit stream employs a hybrid chaotic system, which is employed to encrypt or decrypt the transmitted image and is utilized in the diffusion process to dissipate the redundancy in the original transmitted image statistics. The transmitted image is divided into eight scrambled matrices according to the position of the pixel in every splitting matrix. Each binary matrix is converted using a different conversion rule in the Watson–Crick rules. The DNA confusion stage is applied to increase the complexity of the correlation between the transmitted image and the utilized key. These stages allow the proposed image encryption scheme to be more robust against chosen/known plaintext attacks, differential attacks, cipher image attacks, and information entropy. The system was revealed to be more sensitive against minimal change in the generated secret key. The analysis proves that the system has superior statistical properties, bulkier key space, better plain text sensitivity, and improved key sensitivity compared with former schemes.

## 1. Introduction

Securing multimedia communications is a very important process in modern communication networks [1,2,3,4,5]. Recently, various researches have been dedicated their efforts to developing several schemes to fortify personal digital images. Digital images have many discriminative properties, for instance a wide spectrum and high correlation within the neighboring pixels. These properties make some conventional data encryption schemes not convenient for securing the processing of such information. Therefore, new schemes and approaches, such as deoxyribonucleic acid (DNA) [6,7,8] and chaotic maps [9,10] have been utilized in modern digital image safeguarding schemes. These approaches help to improve robustness against chosen/known plaintext attacks, enriched statistical properties, enhanced key space, amended plain text sensitivity, and upgraded key sensitivity than earlier schemes.

In [11], Qiang et al. introduced a scheme that depended on a DNA series matrix and chaos using dual logistic maps to execute a pair process to the DNA sequences. Following [12], Zhang et al. suggested a new proposal that encoded the plaintext into American Standard Code for Information Interchange (ASCII) code and then converted it into DNA sequential data which will be combined using Exclusive OR (XOR) operation and chaotic map. Subsequently, in [13], Wei et al. developed a new image encryption process reliant on DNA, chaotic images combination, and utilized hamming distance to generate a robust crypto-keys. Then in [14], Rasul et al. recommended an encryption system with the aid of chaos function accompanied by a modified genetic algorithm, and DNA structure is employed for more security. Liu et al. [15] proposed an algorithm depending on a DNA rule merged with chaotic map. In addition, they constructed a cipher procedure based on a one-time encryption key and the chaotic maps to improve the safety and the dynamic deprivation of the proposed procedure, where the primary conditions are created by the MD5 of mouse positions. Later in 2014, Yushu et al. [16] examined the role of image fusion, which depends on crypto-analysis for the encryption method and hiding the information using DNA classification combined with the chaotic map for covering the information [17]. In 2015, Zhang et al. proposed that an image cryptosystem depends on the lookup table concept. They built a different cipher for a digital image cryptosystem that depends on the Latin square and chaos theories [18]. Following in 2017, an efficient digital image encryption process utilizing adaptive rearrangement diffusion and an arbitrary DNA coding is presented [19]. Haider et al. in 2017 [20] proposed a different hybrid encryption algorithm that employs triangular scrambling where DNA mapping and the chaotic map was utilized to increase the security of the scheme. These articles have no robustness against noise, and their conclusions appeared to have a relatively small local and global entropy of the encoded image. On the other hand, the number of pixel change-rates (NPCRs) and the unified average changing intensity (UACI) of the resulted cipher image is far off from the proofed theoretical values, which means that these procedures are not receptive enough to small variations of the input image.

A perfect cryptosystem must satisfy some performance analysis such as large key space, sensitivity to slight change in the secret key, and nearly no correlation between two consecutive adjacent pixels [21]. In 2019, Chengqing Li et al. [22] solving scenario-oriented image security problems by introducing an algorithm with new technologies. In the same year, Zhongyun et al. proposed a cryptosystem that depends on the principles of the Josephus problem and the filtering tools. The algorithm utilizes a standard diffusion and confusion configuration [23]. In [24], a new color image scheme with energetic DNA and a 4D hyperchaotic system is proposed that satisfies the above requirements.

In this article, a new secured encryption algorithm is presented to encrypt an image utilizing an identical encryption and decryption schemes to improve the performance and the security analysis by employing a genome scrambling stage that depends on the DNA mutation process to be robust against several attacks. The plain image is divided into eight scrambled matrices according to the position of the pixel in every splitting matrix. Then, each binary matrix of the eight scrambled matrices is converted using a different conversion rule in the Watson–Crick rules. For example, we scrambled the first matrix with rule 1, the second matrix with rule 2, and so on. This mutation process increases the complexity of the relationship between the transmitted image and the utilized key and allows the proposed encryption algorithm to be more robust against chosen/known plaintext attacks.

The presented algorithm is given in an accurate mathematical language with no exceptional elements and tested against the list given in [25]. It is a bijective algorithm, and its key space is evaluated and tested. The two diffusion processes are presented in mathematical prepositions and the DNA diffusion stage is well defined with numerical examples. The procedure follows Kerckhoffs’ principles, as it does not comprehend any stealthy factors except for the key. It also follows Shannon’s two primitive proposes as it contains two non-consecutive diffusion stages employing the hybrid chaotic map and one confusion stage between them via DNA. From the numerical analysis, it passes many statistical and randomness tests such as histogram analysis, NPCR, UACI, and various correlation tests, and its scores are better than previously presented schemes in most of the tests. The proposed encryption and decryption are more robust against brute force, differential cipher images, and entropy attacks than previous schemes. It has a bigger key space and it is more sensitive to minimal change in the chosen secret key than former techniques.

The remainder of the article is structured as follows. Section 2 details the related background for the employed chaotic map and the DNA cryptography approach. Following is the explanation and discussion of the proposed encryption/decryption procedure in Section 3. Section 4 gives the numerical simulation results, while the security performance of the presented scheme is analyzed in Section 5. Finally, Section 6 presents the whole presented work and conclusions.

## 2. Related Background

### 2.1. Employed Chaotic System

Chaotic cryptography is an essential tool to develop fortified encryption schemes that improve the security analysis performance of cryptographic algorithms where the distinct chaotic assets such as the nature of determinism, random performance, nonlinear conversion, sensitivity to preliminary conditions, and structure parameters have approved “chaos” as an encouraging substitute for conventional and public key cryptographic algorithms. Since Matthews employed chaos into cryptology for the first time in 1989 [26], many chaotic systems have been employed in image encryption [27,28,29]. In 1963, Lorenz [30] discovered the three-dimensional (3D) independent chaotic system. Other chaotic systems introduced in succession include the Chen-Lu chaos system [31] and Liu chaos system [32]. Both employed the 3D chaos system, which provides only a single positive Lyapunov exponent (PLE). However, the chaotic system with multiple PLE improves the vibrant behaviors of such a structure, making it more complicated and difficult to predict. Recently, the adaptive control methods for the four-dimensional (4D) chaotic systems were introduced in [33,34], which are stiffer to expect than previous systems. In different chaotic image encryption schemes, the chaotic map initial values or parameters are employed as private keys, and the iterations of chaotic systems are operated as a producer of pseudo-random sequences that convert original images into noise-like encrypted cipher images. These systems behave as non-periodic in state space due to their sensitivity to the chaotic parameters and chaos initial conditions. Wang et al. presented the perceptual conception of artificial neural network into the chaotic system and proposed that an image chaotic encryption algorithm relied on perceptron [35]. In addition, they [36,37] have reported the hyper-chaos Lorenz system as

(1) $$\begin{array}{l} \frac{dx}{dt}=a(y-x)+w,\\ \frac{dy}{dt}=cx-y-xz,\\ \frac{dz}{dt}=xy-bz,\\ \frac{dw}{dt}=-rw-yz, \end{array}$$

where *b* > 0, *a* > 0, *r* > 0, and *c* > 0 are the constraints of the Lorenz hyper-chaos structure which determine the chaotic behaviors and bifurcation of the hyper-chaos Lorenz map. When *b* = 8/3, *a* = 10, *c* = 28, and *r* = 1, the system behaves in a hyper-chaotic manner. The actual ranges of primary variables are as follows: $w_{0}\in (-\;250,\;250)$, $y_{0}\in (-\;40,\;40),\;z_{0}\in \left(1,\;81\right)$ and $x_{0}\in (-\;40,\;40),\;$ which are always considered as part of the chosen secret key. The step size is equal to 2 × 10^−3^ when digitizing (1) by the Runge Kutta method in the fourth order [38].

### 2.2. DNA Cryptography

DNA cryptography is a promptly emerging technology that depends on theories of DNA structures and is known as a promising technology for unbreakable robust algorithms. It is defined as hiding data in terms of DNA sequences and is used in transmitting or storing data. The DNA is the genetic material in living organisms, which includes all the essential information to construct and sustain it. The strands of DNA are long polymers of several units named nucleotides. The nitrogen base consists of quad nucleic acids: Adenine (A), Cytosine (C), Guanine (G), and Thymine (T). The DNA has a dual helix arrangement formed by coupling dual chains of nucleic acids all together. Every chain is a complement to the other one; T and A are paired duos, while C and G are alternative paired duos. The 0 and 1 are a complement pair in a binary operation; thus, 0 0 and 1 1 are a complement pair, and 0 1 and 1 0 are another complement pair. For example, the A, T, G, and C nucleic acid bases can be encoded as 0 0, 1 1, 1 0, and 0 1, correspondingly. Table 1 indicates the utilized DNA eight possible encoding guidelines that satisfy complementary rubrics [39].

### 2.3. Traditional Chaotic Encryption System

**Figure 1:**
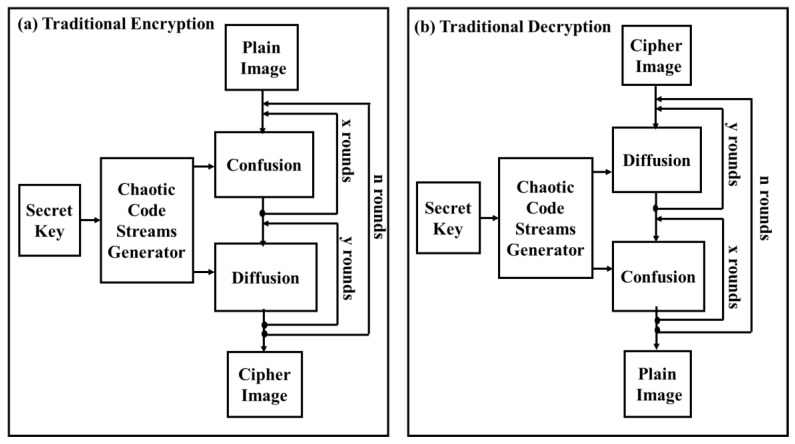
An example of image securing scheme that depends on a chaotic map: (**a**) traditional encryption, (**b**) traditional decryption.

The traditional chaotic image encryption algorithm depends on x repeated confusion stages and y repeated diffusion stages, and both of these stages are reiterated n periods to generate the ciphered image from the original plain one, as shown in Figure 1a. To recuperate the plain image from the ciphered one, the chaotic decryption algorithm is applied and it is the contrary of the encryption algorithm presented in Figure 1b.

## 3. Proposed Image Cryptosystem

**Figure 2:**
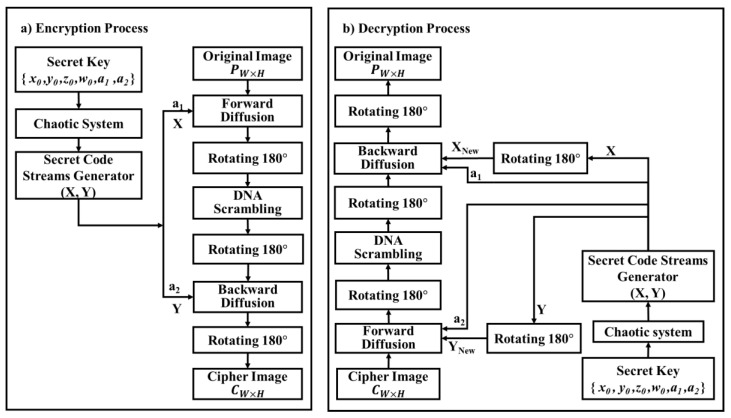
The proposed securing system for images: (**a**) encryption process, (**b**) decryption process.

Let **P** represent the originally transmitted image, which is exemplified by a matrix with size *W × H* where *W* and *H* are the size of the columns and rows of the matrix **P**, respectively. Each element of the grayscale original image is signified by 8-bit i.e., 256 intensity levels. The presented encryption scheme is illustrated in Figure 2. Its private key is defined as $S=\{x_{0},\;y_{0},\;z_{0},\;w_{0},\;a_{1},\;a_{2}\}$, where $y_{0},x_{0},\;z_{0},$ and $w_{0}$ are the initial parameters of the 4D hyperchaotic system, whose value ranges are $y_{0}\in (-\;40,\;40),\;x_{0}\in (-\;40,\;40),\;w_{0}\in (-\;250,\;250),\;\mathrm{and}\;z_{0}\in \left(1,\;81\right)$ where $a_{1}$ and $a_{2}$ are 8-bit random numbers selected by the user. The step size of $x_{0},\;y_{0},\;z_{0}$ is $10^{-13}$, while the step size of $w_{0}$ is 10^−12^. In the encryption process, there are dual pseudo-random matrices **X** and **Y** that are produced by repeating the hyperchaotic system to encrypt the original image. In the decryption algorithm, the pseudorandom matrices are produced by the indistinguishable methods utilized in the encryption algorithm; then, these matrices are rotated a half cycle to generate new different matrices designated by **Y_New_** and **X_New_**.

In the presented scheme, the encryption and decryption algorithm are the same, both counting the exact identical stages of the forward image diffusion stage, circling the resulting image by 180 degrees, scrambling the outcome with the DNA confusion stage, rotating the consequence by 180 degrees, utilizing the backward image diffusion, and rotating it by 180 degrees again. The algorithm comprises four main stages, as portrayed in the subsequent sections.

### 3.1. Secret Code Stream Generator

There are two pseudo-random matrices are generated using the hyperchaotic Lorenz system given by Equation (1), represented by **X** and **Y,** and both of the size *W × H.* Iterate this equation beginning with the four initial values defined in the private key S for $a_{1}+a_{2}$ times in order to avoid the ephemeral values of the hyperchaotic Lorenz system, and then resume iterating for *W × H* to get four pseudo-noise streams, which are designated by {*x_i_*}, {*y*_i_}, {*z*_i_}, and {*w*_i_}, *i* = 1, 2, ···, *W × H*, separately. Generate the two matrices **X**, **Y** from the sequences {*x_i_*}, {*y*_i_} by

(2) $$X(k,l)=Floor((x_{(k-\;1)\;\times H+l}+500\mathrm{mod}1)\times 10^{13})\mathrm{mod}256,$$

(3) $$Y(k,l)=Floor((y_{(k-\;1)\;\times H+l}+500\mathrm{mod}1)\times 10^{13})\mathrm{mod}256,$$

where *Floor(f)* yields the largest principle integer number not as much of *f*, and “+500” is used to translate any negative numbers to positive numbers. These two matrices are used for forward and backward diffusion in the encryption process. However, for the decryption procedure demonstrated in Figure 2b, new matrices **X_New_** and **Y_New_** are generated by spinning the novel matrices **X** and **Y** by 180 degrees, correspondingly. They are generated utilizing

(4) $$X_{New}(i,\;j)=X(i,\;W+1-j),\;for\;i=1\ldots H,\;j=1\ldots W,\;$$

(5) $$Y_{New}(i,\;j)=Y(i,\;W+1-j),\;for\;i=1\ldots H,\;j=1\ldots W.$$

### 3.2. Forward Diffusion Stage

In this stage, the algorithm obtains a new matrix denoted by **Q** by applying XOR (⊕) operation between the two matrices of the original image **P** elements and the pseudo-random matrix X elements according to the subsequent formulas

(6) $$Q(1,1)=P(1,1)⊕X(1,1)⊕a_{1}$$

(7) Q(1,j)=P(1,j)⊕X(1,j)⊕Q(1,j−1), for j=2,3,…,W

(8) Q(i, 1)=P(i, 1)⊕X(i, 1)⊕Q(i−1, 1), for i=2,3,…,H

(9) $$\begin{array}{c} Q(i,\;j)=P(i,\;j)⊕X(i,\;j)⊕Q(i-1,\;j)\;⊕\;Q(i,\;j-1)⊕Q(i-1,\;j-1),\;\\ \;for\;i=2,3,\ldots ,H,\;and\;j=2,3,\ldots ,W \end{array}$$

Then, we rotate matrix **Q** by 180 degrees to attain a matrix designated by **A** using

(10) A(i, j)=Q(i, W+1−j), for i=1,2...H, and j=1,2...W

which is the input to the next DNA scrambling stage described in the following subsection.

### 3.3. DNA Mutation Scrambling Stage

To improve the fight of the encryption process against chosen/known plaintext attacks, we introduce a new scrambling stage that depends on the DNA mutation process. Utilizing this stage improves the complex relative between the plain image, the encrypted one, and the utilized key. A small difference in the original image or key will affect a major divergence in the encrypted image with assent. The steps to get the scrambled matrix are as follows.

**Figure 3:**
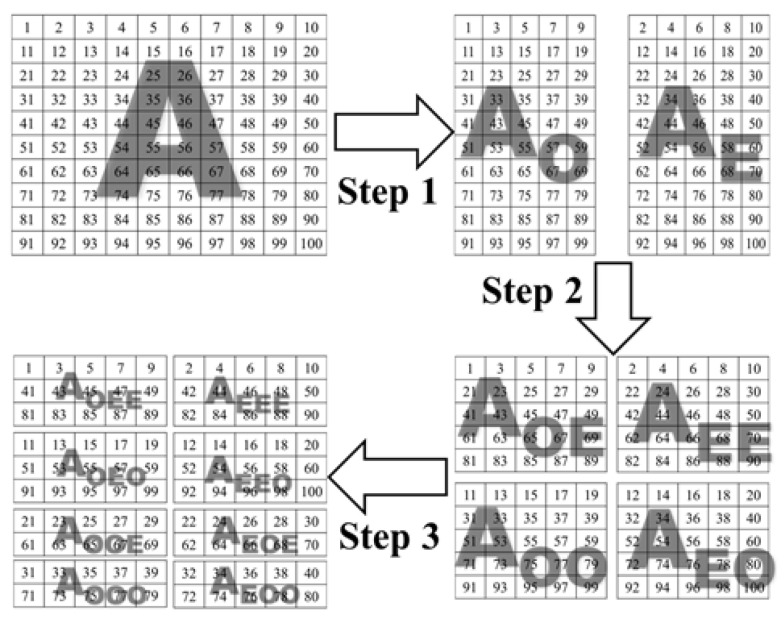
The effect of applying the first three steps of the DNA mutation scrambling stage on a 10 × 10 matrix.

1. Divide matrix **A** into two equally matrices by selecting the even columns together and create the first matrix **A**_E_; then, select the odd columns and create the second matrix **A**_O_. Both matrices have the size of H×W/2.
2. Generate two new matrices **A**_OE_ and **A**_OO_ from **A**_O_ by selecting the even rows together and the odd rows together. Repeat for **A**_E_ to generate **A**_EE_, **A**_EO_ matrices. The four matrices have the size W/2×H/2.
3. Repeat Step 2 to generate eight new matrices **A**_EEE_, **A**_EEO_, **A**_EOE_, **A**_EOO_, **A**_OEE_, **A**_OEO_, **A**_OOE_ each of size W/2×H/4. Figure 3 illustrates the output of every step applied to matrix **A** with a size of 10 × 10.
4. Convert each pixel into the eight matrices to 8-bit binary values, each of size 4W×H/4. For example, the first three elements in matrix A_OOO_ are ‘31’, ‘33’, and ‘35’, which will be converted to ‘00011111’, ‘00100001’, and ‘00100011’.
5. Encode every binary element in the eight matrices with the DNA encoding rules. Each binary matrix is encoded using a different conversion rule, as shown in Table 1. For instance, rule 1 is used to encode the first matrix, rule 2 is used for the second matrix, etc. The outputs of this stage are eight DNA encoded matrices with the size of 2W×H/4. For illustration, ‘00011111’, ‘00100001’, and ‘00100011’ will be encoded with Rule 8 to ‘GACC’, ‘GTGA’, and ‘GTGC’, respectively.
6. Mutate the existing DNA pairs to their mutated values according to Table 2. For illustration, the ‘GACC’, ‘GTGA’, and ‘GTGC’ will be mutated to ‘CAGC’, ‘CTCA’, and ‘CTCC’, respectively.
7. Convert the mutated DNA values to their corresponding binary values according to Table 1. For example, ‘CAGC’, ‘CTCA’, and ‘CTCC’ will be mutated to ‘11010011’, ‘11101101’, and ‘11101111’, correspondingly.
8. Concert the binary values to their decimal values in the range from ‘0’ to ‘255’. For instance, the ‘11010011’, ‘11101101’, and ‘11101111’ will be converted to 211, 237, and 239, respectively.
9. Concatenate the eight matrices into one decimal matrix denoted by I of size W×H with the new confused values and rotated by 180 degrees to generate a matrix denoted by **B** fed to the next stage of backward diffusion.

### 3.4. Backward Diffusion Stage

In the backward image diffusion stage, the matrix **B** is converted into a matrix signified by **E**, with the XOR process and the pseudo-noise matrix **Y** by

(11) $$E(H,W)=B(H,W)⊕Y(H,W)⊕a_{1}$$

(12) E(H,j)=B(H,j)⊕Y(H,j)⊕B(H,j+1), for j=W−1,…,1

(13) E(i,W)=B(i,W)⊕Y(i,W)⊕B(i+1,1), for i=H−1,…,1

(14) $$\begin{array}{c} E(i,j)=B(i,j)⊕Y(i,j)⊕B(i+1,j)⊕B(i,j+1)⊕B(i+1,j+1),\\ \;for\;i=H-1,\;\ldots ,1,\;for\;j=W-1\;,\;\ldots ,1 \end{array}$$

Turn around the matrix **E** by 180 degrees to get a ciphered image matrix symbolized by **C** using

(15) C(i,j)=E(i,W+1−j), for i=1,2...H,and j=1,2...W

## 4. Simulation Effects

**Figure 4:**
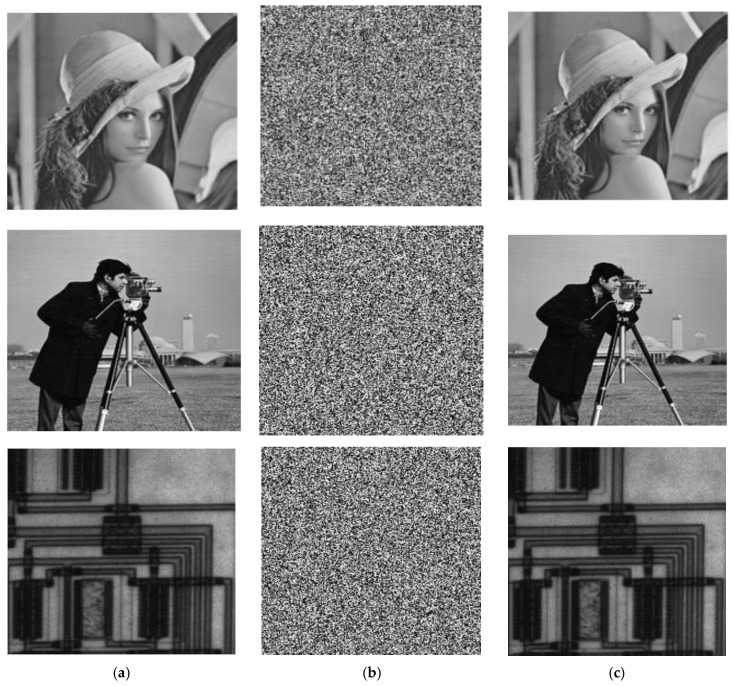
Simulation results. (**a**) plain images, (**b**) Generated cipher images of (a), (**c**) Recovered image from (b).

**Figure 5:**
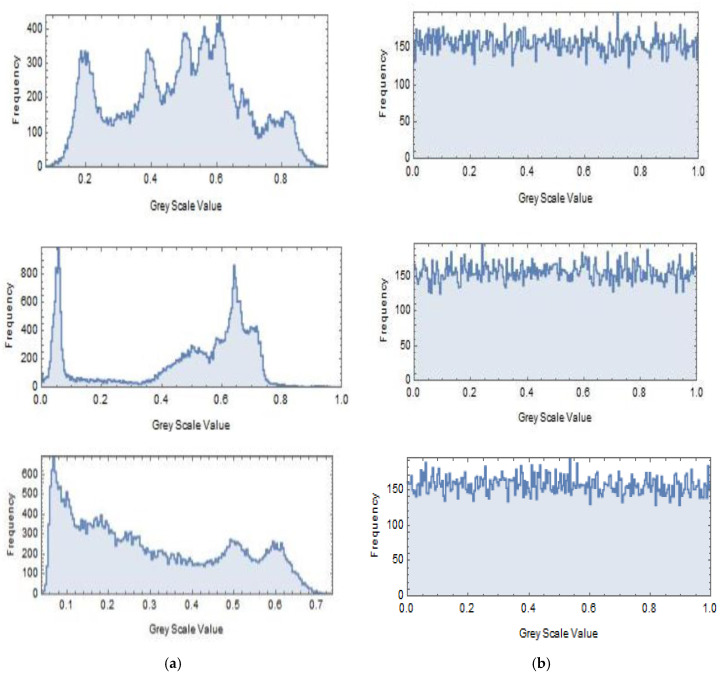
Histograms analysis. (**a**) Histograms of plain images, (**b**) Histograms of cipher images.

The algorithm is carried out on the Lena, Cameraman, and Circuit images. ‘Mathematica 11’ software is used to apply the proposed symmetric encryption and decryption scheme. The utilized secret symmetric key is set to *S* = {3.3133, 12.0546, 40.8879, −34.5677, 35, 201}, which is employed in each encryption and decryption algorithm, and the used plain images with size 200 *×* 200 pixels are illustrated in Figure 4a. The corresponding cipher image is generated using the encryption algorithm, and it is shown in Figure 4b. Then, we decrypted the cipher image Figure 4b with the correct secret key *S* to get the perfectly reconstructed image, as shown in Figure 4c. The histograms of the plain images and the corresponding generated cipher images are shown in Figure 5a,b, respectively. It’s clear from Figure 4 and Figure 5 that the pattern of the cipher image is noise-like and is not related to the original plain image. The recovered image is a perfectly reconstructed version of the original plain image, while the histogram of the cipher image is almost flat, which ensures that no statistical information from the original image can be discovered consequently. 

## 5. Performance and Security Analysis

### 5.1. Key Space

The key space is usually designed to be large enough to prevent an opponent from using a brute-force attack to find the key used to encrypt the plain images. In this article, the key space of the presented cryptosystem consisted of the initial conditions of the hyperchaotic Lorenz system $x_{0},y_{0},z_{0},w_{0},a_{1}\;\mathrm{and}\;a_{2}$. The value ranges were $x_{0}\in (-\;40,40)$, $y_{0}\in (-40,40)$, and $z_{0}\in (1,80)$ each with a step size of $10^{-13}$, while the value range of $w_{0}\in (-250,250)$ had a step size of $10^{-12}$. $a_{1}$ and $a_{2}$ are two 8-bit random numbers whose value ranges are [0,255] with a single step size. Therefore, the key space of the proposed algorithm is $1.6777\times 10^{64}$, and that space is large enough to resist brute-force attacks. It would take $2.03451\times 10^{21}$ days to crack the systems if 17 billion attempts are tried hourly using a very high-performance machine.

### 5.2. Statistical Attacks Analysis 

The encryption process should have the ability to struggle against statistical confrontation, and this can be assessed by histogram analysis and a chi-square test.

#### 5.2.1. Image Histogram Analysis

**Figure 6:**
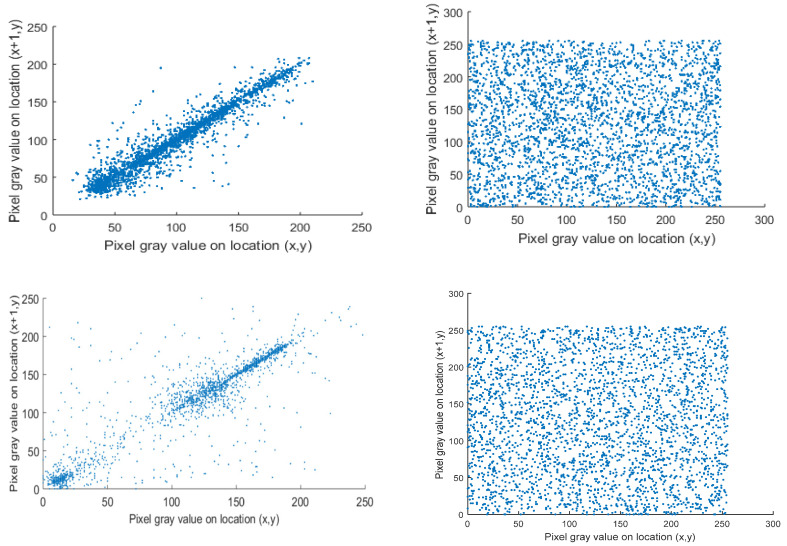

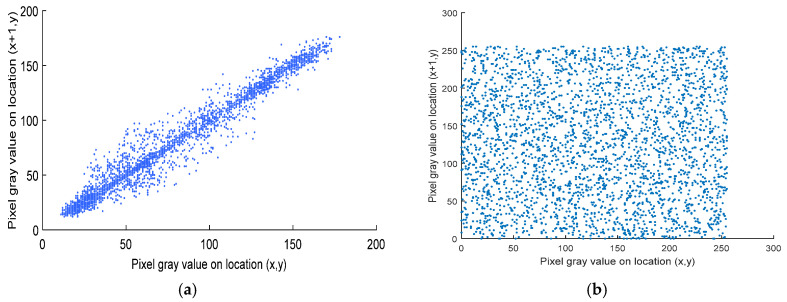
Correlation analysis plot. (**a**) Horizontal direction correlation for plain images Lena, Cameraman, and Circuit, (**b**) Horizontal correlation for their cipher images respectively.

The histograms of the original images of Lena, Cameraman, and Circuit shown in Figure 5a are non-uniform. The characteristic peak is clear and most of the images’ information can be obtained effortlessly. On the other hand, Figure 5b displays the histograms of the cipher images with a nearly uniform statistical distribution. These two aspects prove the statement of resisting the statistical attacks and the cipher images attack in our proposed encryption process. The correlation among nearby pixels signifies the randomness of the encrypted resulted gray levels. Now, we discuss the correlation in the horizontal, the vertical and the diagonal directions. Choose a random *N* duos of nearby pixels, and $\left(x_{i},y_{i}\right)$ are the concerned pixel values of the *i*-th pair (*i* = 1, 2, …, *N*). After that, the correlation coefficient *r* can be computed by

(16) $$r=\frac{\sum_{i=1}^{N}\left(x_{i}-\bar{x})(y_{i}-\bar{y}\right)}{\sqrt{\sum_{i=1}^{N}{\left(x_{i}-\bar{x}\right)}^{2}}\sqrt{\sum_{i=1}^{N}{\left(y_{i}-\bar{y}\right)}^{2}}}$$

where *r* is the sample size, $\left(x_{i},y_{i}\right)$ are the distinct sample points with index *I*, $\bar{x}=1/N\sum_{i=1}^{N}x_{i}$ is the sample mean, and similarly for $\bar{y}$. Now, let *N* = 2000, and the resulted different correlation coefficients of the cipher image and original image are recorded in Table 3. The correlation in the horizontal course for each image is shown in Figure 6. The correlation of plain images is high and close to 1. In contrast, the correlation of the cipher images is low and near to 0, which indicates that encrypted pixels are valued and distributed randomly in the encrypted images.

#### 5.2.2. Chi-Square Test

To proof the uniform distribution of the resulted ciphered images in a more precise manner, we perform the chi-square test to show that the cipher image is a uniform distribution. The chi-square test is described

(17) $$\chi^{2}=\sum_{i=0}^{L-1}\frac{{\left(o_{i}-e_{i}\right)}^{2}}{e_{i}}$$

where *L* is the number of pixel grayscale levels, $o_{i}$ is the occurrence frequency of each gray level (0–255) in the histogram of the resulted encrypted images, and $e_{i}$ is the probable frequency of the uniform distribution. The uniform distribution of the histogram is assessed with the aid of the chi-square $\chi^{2}$ test. The null hypothesis is only accepted when the *p-*value is greater than the significance amount s(s∈[0,1]). Table 4 presents the chi-square score and their *p*-value for the histogram of the encrypted Lena, Cameraman, and Circuit images and the significance level amount of 0.05. The obtained score is smaller than $\chi_{th}^{2}$ (255, 0.05) = 293.247, while their *p*-value is larger than 0.05. Therefore, the null hypothesis is achieved, and the histogram of the encrypted images is uniformly distributed. Based on that, the presented encryption algorithm is strong against statistical attacks.

### 5.3. Key Sensitivity Analysis

To test the key sensitivity of a secret key, we can create a different secret key *S*_2_ after the secret key $S=\{x_{0},y_{0},z_{0},w_{0},a_{1},a_{2}\}$ by changing $\left\{a_{1},a_{2}\right\}$ by 1 or any component of $\left\{x_{0},y_{0},z_{0}\right\}$ by $10^{-13}$ or $w_{0}$ by $10^{-12}$. First, encrypt the plaint image **P** for the presented encryption system with the secret keys of *S*_1_ and *S*_2_ to get dual cipher images, symbolized by **C**_1_ and **C**_2_, correspondingly. Contrast **C**_1_ and **C**_2_ to get two gauges named *Diff*_1_ and *Diff*_2_ using [40]

(18) $$Diff_{1}=\left(\frac{1}{W\times H}\right)\sum_{i=1}^{W}\sum_{j=1}^{H}\left|Sign\;(C_{1}(i,j)-C_{2}(i,j))\right|\times 100\%$$

(19) $$Diff_{2}=\left(\frac{1}{W\times H}\right)\sum_{i=1}^{W}\sum_{j=1}^{H}\frac{\left|C_{1}(i,j)-C_{2}(i,j)\right|}{256}\times 100\%$$

where *Sign(·)* is the sign role, and (*W*, *H*) are the width and height of the original image, correspondingly. Correspondingly, the hypothetical values for $Diff_{1}$ and $Diff_{2}$ in the case of dual arbitrary images are 99.6094% and 33.3328%.

Another way to assess the secret key sensitivity and secure the plain image **P**_1_ utilizing the secret key *S*_1_ of the presented system to get the ciphered image **C**, and then decrypt the ciphered image **C** by the new secret key *S*_2_ to retrieve the plain image signified by **P**_2_. Compare **P**_1_ and **P**_2_ to get $Diff_{3}$ and $Diff_{4}$ respectively, using [40]

(20) $$Diff_{3}=\left(\frac{1}{W\times H}\right)\sum_{i=1}^{W}\sum_{j=1}^{H}\left|Sign\;(P_{1}(i,j)-P_{2}(i,j))\right|\times 100\%$$

(21) $$Diff_{4}=\left(\frac{1}{W\times H}\right)\sum_{i=1}^{W}\sum_{j=1}^{H}\frac{\left|P_{1}(i,j)-P_{2}(i,j)\right|}{256}\times 100\%.$$

Assume that the plain image denoted by **P**_1_ and another random image is denoted by **P**_2_; then, the hypothetical values of $Diff_{3}$ and $Diff_{4}$ are 99.6094% and 28.5059%, respectively [40]. To test the plaintext sensitivity, 100 trials are done for the Lena, Cameraman, and Circuit images and calculate the average values of $Diff_{1}$, $Diff_{2}$, $Diff_{3}$, and $Diff_{4}$. Table 5 shows that the calculated results of $Diff_{1}$, $Diff_{2}$, $Diff_{3}$, and $Diff_{4}$ are nearly equal to their hypothetical values; these values indicators are designating that the presented scheme is very sensitive to minimal alteration in the generated secret key.

### 5.4. Differential Attack

An opponent can get valuable information by altering several pixels of the plain image. The NPCR and UACI are usually employed to measure the resistance of the encrypted image against differential raids. We utilize the presented encryption scheme to encrypt **P**_1_ and **P**_2_ to get their corresponding cipher images denoted by **C**_1_ and **C**_2_ with the same secret key, where **P**_2_ (i, j) = [**P**_1_ (i, j) + 1] mod 256. Then, the NPCR and UACI are given by

(22) $$\mathrm{NPCR}=\frac{1}{W\times H}\sum_{i=1}^{W}\sum_{j=1}^{H}\left|Sign(C_{1}(i,j)-C_{2}(i,j))\right|\times 100\%$$

(23) $$\mathrm{UACI}=\frac{1}{W\times H}\sum_{i=1}^{W}\sum_{j=1}^{H}\frac{\left|Sign(C_{1}(i,j)-C_{2}(i,j))\right|}{256}\times 100\%$$

The theoretical values of NPCR and UACI for any two random images with 256 gray levels are 99.609% and 33.464%, respectively. The NPCR and UACI test outcomes are presented in Table 6 and Table 7, respectively. If one bit of the input image is altered, the NPCR and UACI of traditional methods, in CCAES [41], CDCP [42], and CHC [43] are close to hypothetical values. DNA-based methods, C-DNA [44] and HC-DNA [45], have a better noise attack performance than the previous works. Furthermore, the values are compared against the critical values as in [46,47]. These demonstrate the capability of the proposed algorithm to stand for differential attacks. So, the presented cryptosystem attains high performance by getting NPCR values and UACI values near to their hypothetical values. 

### 5.5. Cipher Image Sensitivity Analysis

To measure the differential attacks based on ciphered image analysis, we obtain the cipher image **C**_1_ by encrypting the plain image **P**_1_ using the corresponding secret key *S*_1_ Then, generate a new cipher image **C**_2_ from **C**_1_ by shifting the randomly selected pixel of the $C_{1}(i,j)$ value by 1 where $C_{2}(i,j)=[C_{1}(i,j)+1]\mathrm{mod}256$ for a selected pixel position (i,j) in a random manner. To analyze cipher image sensitivity firstly, decrypt the ciphered image **C**_2_ utilizing the presented algorithm with the secret key *S*_1_ to get **P**_2_, which is the recovered image. Analyze the difference between the two recovered images **P**_1_ and **P**_2_ by using Equations (15) and (16) to calculate the values of $Diff_{3}$ and $Diff_{4}$. Secondly, change any element of the secret key *S*_1_ to get new secret key *S*_2_, and decrypt **C**_1_ and **C**_2_ by the proposed scheme with the new secret key *S*_2_ to obtain their corresponding recovered images **P**_3_ and **P**_4_, correspondingly. Later, compute the indicators named $Diff_{3}$ and $Diff_{4}$ among **P**_3_ and **P**_4_. Finally, reiterate the overhead steps 100 times and compute the middling values. The calculated value of $Diff_{3}$ equal to 99.6150 and $Diff_{4}$ equals to 28.4170, which are near its hypothetical values. Therefore, the presented scheme is shown to be very vulnerable to the slight alteration of cipher images and can withstand the differential cipher images attacks.

### 5.6. Resisting Chosen/Known Plaintext Attacks

The chosen/known plaintext attack [48,49] can occur when an attacker chooses an arbitrary plaintext and its corresponding cipher text to distinguish the algorithm or the secret key, which allows it to decrypt any cipher text using this algorithm. Assume that dual plain images **P**_1_ and **P**_2_ are identical except for **P**_1_ (i, j) ≠ **P**_2_ (i, j) where **P**_2_ (i, j) = [**P**_1_ (i, j) + 1] mod 256. In the forward image diffusion stage, the plain images **P**_1_ and **P**_2_ are transformed to matrices denoted by **Q**_1_ and **Q**_2_ with the XOR logic process and the pseudo-noise matrix X. Consequently, rotate **Q**_1_ and **Q**_2_ by 180 degrees to get **A**_1_ and **A**_2_. Thus, the pixel located at (1, 1) swaps its position with the pixel located at (*w*_1_, *h*_1_) in **A**_1_, while the pixel located at location (1, 1) swaps its position with the pixel located at (*w*_2_, *h*_2_) in **A**_2_. After one time of DNA scrambling operation, on the pixel (1, 1), the value of **I**_1_ (1, 1) ≠ **I**_2_ (1, 1). In a similar way, the value of **I**_1_ (1, 2) ≠ **I**_2_ (1, 2). This means that as for images **I**_1_ and **I**_2_, we have **I**_1_ (i, j) ≠ **I**_2_ (i, j). Rotate **I**_1_ and **I**_2_ by 180 degrees to obtain **B**_1_ and **B**_2_, respectively; then, we get **B**_1_ (i, j) ≠ **B**_2_ (i, j) for i = 1, 2, *· · ·*, *W*, j = 1, 2, *· · ·*, *H* and **B**_1_ (W, 1) ≠ **B**_2_ (W, 1). According to Figure 2, by backward diffusion operation, **E**_1_ and **E**_2_ are generated from **B**_1_ and **B**_2_ respectively, so according to the proposed algorithm described, we can get **E**_1_ (i, j) ≠ **E**_2_ (i, j). Thus, **C**_1_ (i, j) ≠ **C**_2_ (i, j), i = 1, 2, *· · ·*, *W*, j = 1, 2, *· · ·*, *H*. These revealed that for identical plain images **P**_1_ and **P**_2_ with just one pair of pixels being dissimilar, their ciphered images **C**_1_ and **C**_2_ will be unique for each corresponding pixel location, even when they are cyphered with an identical secret key. Therefore, the presented encryption algorithm can withstand the chosen/known plaintext attacks.

### 5.7. Global Information Entropy

The global information entropy indicates the uncertainty of global image information, which is denoted by H(m) of matrix *m*, and evaluated as

(24) $$H(m)=-\sum_{i=0}^{255}p(m_{i})\log_{2}((p(m_{i}))$$

where $p(m_{i})$ represents the probability of $m_{i}$. The theoretical value of the global information entropy intended for an 8-bit grayscale random image is nearer to 8. The global information entropy calculated results are presented in Table 8. We take the plain image Lena and the corresponding ciphered image as an example with a grayscale level *L* of 256. In the proposed system, the information entropy of the plain image is equal to 7.4430 and the entropy of the cipher image is equal to 7.9882, which demonstrates that the proposed cryptosystem can resist entropy attacks efficiently.

### 5.8. Local Shannon Entropy

Wu et al. [50] have expressed a new entropy gauge to regulate the real randomness by picking the non-overlapping blocks inside the encrypted image. The local Shannon entropy (LSE) is computed by calculating the mean of several global Shannon entropies on every one of the building blocks. Taking into account the randomness of the ciphered image, but the global entropy analysis in the previous section, the LSE can be defined by

(25) $$H_{k,l}(m)=-\sum_{i=0}^{k}\frac{H(m_{i})}{k}$$

where *m*_1_, *m*_2_, …, *m_k_* are *k* selected image blocks, while *l* is the amount of pixels for each block. The local entropy values for the encrypted image are presented in Table 9. It can be shown that the local entropy value is nearer to the optimum hypothetical value (≈8). Therefore, the proposed algorithm has good randomness.

### 5.9. Complexity Analysis

To compute the complications of performing the presented algorithm, the image size as *W × H* is taken into consideration. Let *n* indicate the quantity of pixels inside the image. The complexity of the presented algorithm can be determined by the following discussed operations. These operations consist of binary data conversion, DNA scrambling operation, secret key generation, forward and backward image diffusion, and decimal data conversion. The complexity of binary data conversion is O(*n*^2^) and that of the DNA scrambling operation is equal to O(4*n*^2^). The secret key creation process consists of tri-sub-operations such as pseudo-random sequence production, binary transformation, and DNA scrambling with a complexity of O(6*n*^2^). In contrast, the complexity of forward and backward diffusion operations is O(62*n*^2^). The conversions from DNA to binary data and binary data to decimal data take O(5*n*^2^). Therefore, the overall complexity of the presented image encryption scheme is O(78*n*^2^).

### 5.10. Encryption and Decryption Speed

The computer used was constructed with Intel Duo Core I7 M460@2.53 GHz, 8 GB DDR3 RAM, Windows 10. For the encryption time *T_E_* and decryption time *T_D_*, the effect of $a_{1}+a_{2}$ can be neglected due to its very small value contrasted to the effect of the original image size. So, we put the values of $a_{1}$ and $a_{2}$ to 128. We made the experiments on the pictures with the size of 200 *×* 200 pixels and recorded the encryption/decryption algorithm duration in Table 10.

From Table 10, we can see that for 1000 pieces of images encrypting or decrypting, the execution times of different encryption schemes was listed. As can be seen, the encryption speed of our proposed algorithm is sufficiently fast to meet real-time performance necessities.

## 6. Conclusions

This article introduced a new bijective algorithm which is dedicated to secure image transmission over the data communication systems. Both encryption and decryption algorithms are identical and each have two diffusion processes and one DNA confusion process, which reduces hardware implementation complexity according to Shannon’s’ proposal. The diffusion process employs the hyperchaotic system and the confusion process uses the DNA, which both enhance the proposed algorithm security. When compared with former algorithms, the statistical tests proved that the proposed cryptosystem has a larger key space to resist brute-force attack, and the randomness tests showed that the encrypted pixels are distributed more randomly through the ciphered image. In addition, the proposed system was revealed to be more sensitive than former techniques against minimal change in secret key and better resistance against the known plaintext, the chosen plaintext, the differential cipher image attacks, and entropy attacks. 

Finally, as future research, we advise an additional investigation of the simulation analysis of the chaotic performance by using an exponential chaotic model and other confusion techniques to generate a robust chaotic image encryption technique with the addition of fault-tolerance technology for the purpose of enhanced transmission of high-quality and secure data. 

## Figures and Tables

**Table 1:** Watson–Crick rubrics.

	A	T	C	G
R 01	0 0	1 1	1 0	0 1
R 02	0 0	1 1	0 1	1 0
R 03	1 1	0 0	1 0	0 1
R 04	1 1	0 0	0 1	1 0
R 05	1 0	0 1	0 0	1 1
R 06	0 1	1 0	0 0	1 1
R 07	1 0	0 1	1 1	0 0
R 08	0 1	1 0	1 1	0 0

**Table 2:** DNA mutation rules.

DNA	Mutate	DNA	Mutate	DNA	Mutate	DNA	Mutate
A A	T A	C A	G A	G A	C A	T A	A A
A C	T C	C C	G C	G C	C C	T C	A C
A G	T G	C G	G G	G G	C G	T G	A G
A T	T T	C T	G T	G T	C T	T T	A T

**Table 3:** Calculated correlation coefficients.

Correlation	Plain Image			Cipher Image		
	Horizontal	Vertical	Diagonal	Horizontal	Vertical	Diagonal
Lena	0.9650	0.9144	0.9056	0.0082	−0.0032	−0.0025
Cameraman	0.9560	0.9140	0.9077	0.0074	−0.0029	−0.0019
Circuit	0.9580	0.9230	0.9118	0.0078	−0.0034	−0.0021

**Table 4:** Histogram chi-square test.

Image	*χ*^2^ Test		
	Score	*p-*Value	*H*_0_
Lena	246.804687	0.6834	Accepted
Cameraman	246.817237	0.6670	Accepted
Circuit	246.794528	0.6754	Accepted

**Table 5:** Key sensitivity tests results (%).

Theoretical Values	*Diff*_1_ (99.6094)	*Diff*_2_ (33.3328)	*Diff*_3_ (99.6094)	*Diff*_4_ (28.5059)
Lena	99.6225	33.3962	99.6250	28.3191
Cameraman	99.6122	33.3876	99.6299	28.3245
Circuit	66.6179	33.3471	99.6134	28.3770

**Table 6:** Number of pixel change rat (NPCR) test analysis (%).

Algorithms	NPCR (%)	NPCR Critical Values		
		$N_{0.05}^{*}\;99.5693\%$	$N_{0.01}^{*}\;99.5527\%$	$N_{0.001}^{*}\;99.5341\%$
Proposed	99.6150	Successful	Successful	Successful
CCAES [41]	99.5697	Successful	Successful	Successful
CDCP [42]	100	Successful	Successful	Successful
CHC [43]	99.6605	Successful	Successful	Successful
C-DNA [44]	$15.25\times 10^{-4}$	NA	NA	NA
HC-DNA [45]	59.7406	NA	NA	NA

**Table 7:** Unified average changing intensity (UACI) test analysis (%).

Algorithms	UACI (%)	UACI Critical Values		
		$U_{\pm \;0.05}^{*}$ +33.2824% −33.6447%	$U_{\pm \;0.01}^{*}$ +33.2255% −33.7016%	$U_{\pm \;0.001}^{*}$ +33.1594% −33.7677%
Proposed	33.4205	Successful	Successful	Successful
CCAES [41]	33.4767	Successful	Successful	Successful
CDCP [42]	33.5752	Successful	Successful	Successful
CHC [43]	33.4263	Successful	Successful	Successful
C-DNA [44]	$8.97\times 10^{-6}$	NA	NA	NA
HC-DNA [45]	25.0487	NA	NA	NA

**Table 8:** Information entropy.

Average Performance	Proposed System			CCAES [41]	CDCP [42]	CHC [43]	C-DNA [44]	HC-DNA [45]
	Lena	Cameraman	Circuit					
**Plain**	**7.443**	**7.432**	**7.438**	**7.422**	7.438	7.441	7.428	7.431
Cipher	7.988	7.997	7.995	7.997	7.966	7.997	7.996	7.996

**Table 9:** Local Shannon entropy.

Image	Proposed System	[51]	[52]	[53]	[54]
Lena	7.903462	7.902838	7.900975	7.904512	7.904671

**Table 10:** Encryption/Decryption time.

Algorithms	Proposed System	CCAES [41]	CDCP [42]	CHC [43]	C-DNA [44]	HC-DNA [45]
Time	0.19253	2.9	2.70264	3.17265	2.15572	0.27783
